# Uricase deficiency causes mild and multiple organ injuries in rats

**DOI:** 10.1371/journal.pone.0256594

**Published:** 2021-08-26

**Authors:** Nan Fan, Yun Yu, Lvyu Li, Heng Xia, Xiangxian Dong, Yongkun Li, Huan Chen, Weigang Duan

**Affiliations:** 1 The Department of Pharmacology, School of Basic Medicine, Kunming Medical University, Kunming, Yunnan Province, China; 2 The Third Affiliated Hospital, Yunnan University of Traditional Chinese Medicine, Kunming, Yunnan Province, China; 3 Yunnan Provincial Key Laboratory of Molecular Biology for Sinomedicine, School of Basic Medicine, Yunnan University of Traditional Chinese Medicine, Kunming, Yunnan Province, China; George Washington University School of Medicine and Health Sciences, UNITED STATES

## Abstract

Uricase-deficient rats could be one of the optimal model animals to study hyperuricemia. The present study aimed to find the biological differences between uricase-deficient (Kunming-DY rats) and wild-type male rats. Uricase-deficient rats and wild-type rats were commonly bred. Their body weight, water and food consumption, 24-h urine and feces, uric acid in serum and organs, and serum indexes were recorded or assayed. Organs, including the heart, liver, spleen, lung, kidney, thymus, stomach, duodenum, and ileum, were examined using a routine hematoxylin-eosin staining assay. We found that the growth of male uricase-deficient rats was retarded. These rats excreted more urine than the wild-type rats. Their organ indexes (organ weight body weight ratio), of the heart, liver, kidney, and thymus significantly increased, while those of the stomach and small intestine significantly decreased. The uricase-deficient rats had a significantly higher level of serum uric acid and excreted more uric acid via urine at a higher concentration. Except for the liver, uric acid increased in organs and intestinal juice of uricase-deficient rats. Histological examination of the uricase-deficient rats showed mild injuries to the heart, liver, spleen, lung, kidney, thymus, stomach, duodenum, and ileum. Our results suggest that uricase-deficient rats have a different biological pattern from the wild-type rats. Uricase deficiency causes growth retardation of young male rats and the subsequent increase in serum uric acid results in mild organs injuries, especially in the kidney and liver.

## Introduction

Hyperuricemia is regarded as a vital factor for the development of gout, renal dysfunction, and osteoarthritis [1]. It is also regarded as a risk factor closely correlated with hypertension, type 2 diabetes mellitus, and hyperlipidemia [2, 3]. Previous reports have suggested that hyperuricemia is tangled with the injuries to the intestinal tract [4], liver [5] and heart failure [6]. Hyperuricemia and associated disorders cause a significant economic burden on modern society. The solution to these diseases should be based on an accurate and profound understanding of their underlying mechanism. Because of the well-known limitations of clinical studies, the best way to understand the disorders and associated diseases is to use animal models. So far, most knowledge of hyperuricemia and related conditions has been obtained from model animals expressing uricase. Uricase, also named urate oxidase (Uox), can transform uric acid into 5-hydroxyisourate; other enzymes then convert it into allantoin [7], a more soluble substance than uric acid. However, uricase is also a big obstacle to establish animal hyperuricemia models. To mimic clinical hyperuricemia in animals with uricase, uricase inhibitors such as oxonate [8] or urate excretion inhibitors such as ethambutol [9], have been usually applied.

However, uricase is an inducible enzyme. A high level of serum uric acid (SUA) upregulates the expression of uricase and makes the so-called hyperuricemic model unstable [10]. Additionally, the use of oral medications can be tangled with their other bioactivities, so that made the hyperuricemia model much complex beyond anticipation [11]. Further, false-positive results can be obtained if a drug disturbs the absorption, distribution, metabolism, or excretion of the oral chemicals that were used to establish the hyperurecemia model. It is clear that the use of uricase-deficient animals would easily overcome these shortcomings, and the uricase-deficient (Uox^-/-^) model animal has always been desired by scientists. As early as in 1994, Uox^-/-^ mice were generated by homologous recombination in embryonic stem cells [12]. Recently, Uox^-/-^ mice has been generated using the transcription activator-like effector nuclease (TALEN) technique [13] with the hope to mimic human’s purine metabolism. Uox^-/-^ mice should be an ideal model to study the mechanism of hyperuricemia and associated disorders, but they cannot survive long enough for long-term studies [12, 13]. Last year, an Uox^-/-^ rat (“Kunming–DY” rat) was generated using the CRISPR/Cas9 technique by our research team, and it seemed that these rats would overcome the disadvantages of Uox^-/-^ mice [14]. These animals are apparently healthy and can survive for more than one year, though with mild renal injury. The serum uric acid (SUA) in these rats is significantly increased, to a high level similar to that of normal men, but much lower than that of Uox^-/-^ mice. Therefore, the Uox^-/-^ rats could be the right candidate model animal for studying hyperuricemia and associated disorders.

However, besides SUA and renal function, the biological properties of the Uox^-/-^ rats have not yet been systematically investigated, and there could be hidden injuries in their other organs. The present study systematically investigated the biological properties of the Uox^-/-^ rats to clarify whether these rats may be an alternative model animal to study hyperuricemia and associated diseases.

## Materials and methods

### Materials

Wild-type Sprague Dawley (SD) rats were obtained from Chengdu Dossy Experimental Animals Co., Ltd, Chengdu, China [Certification No. SCXK (Chuan) 2008–24]. Uox^-/-^(Kunming-DY), Uox^+/-^, and wild-type 45-day old rats were provided by the laboratory, as previously described [14].

Uric acid assay kits of the phosphotungstic acid method (Lot: C012-1-1), blood urea nitrogen (BUN) assay kits of the diacetyl monoxime method (Lot: C013-1-1), serum creatinine (Cr) of sarcosine oxidase method (Lot: C011-2-1), and protein assay kits of bicinchoninic acid (BCA) method (Lot: A045-4-2) were purchased from Nanjing Jiancheng Bioengineering Institute (Nanjing, China). Total triglyceride (TG) assay kits of glycerol lipase oxidase (GPO-PAP) method (Lot: A110-1-1), total cholesterol (TC) assay kits of GPO-PAP method (Lot: A111-1-1), low-density lipoprotein (LDL) assay kits (Lot: A113-1-1), high-density lipoprotein (HDL) assay kits (Lot: A112-1-1), serum glucose kits of glucose oxidase method (Lot: F006-1-1), aspartate aminotransferase (AST) assay kits (Lot: C010-2-1), alanine aminotransferase (ALT) assay kits (Lot: C009-2-1), and total bilirubin (Tbil) assay kits (C019-1-1) were also purchased from Nanjing Jiancheng Bioengineering Institute (Nanjing, China). TRIzol Plus RNA Purification kit was purchased from Invitrogen (Carlsbad, CA, USA).

Ultrapure water was produced with a Milli Q water purification system manufactured by EMD Millipore Group (Darmstadt, Germany). The NanoDrop ND-1000 spectrophotometer used for experiments was manufactured by PeqLab (Erlangen, Germany). We used a multiple microplate reader of Infinite 200pro manufactured by Tecan Group (Mannedorf, Switzerland). A fluorescence microscope was manufactured by Olympus Corp. (Tokyo, Japan). Other instruments or reagents used in the present study were made in China if not stated otherwise.

### Animal breeding

Uox^-/-^ and Uox^+/-^ rats were provided by the laboratory, as previously described [14]. Briefly, male Uox^-/-^ rats were mated with female Uox^-/-^ rats for 3 weeks to generate their offspring. The offspring were breastfed to the age of 3 weeks by their mother; afterward, the mothers, the male offspring, and the female offspring were separated and put in three individual cages. When the male offspring were 45 days old, they were included in the study. Uox^+/-^ rats were generated using similar methods; besides, male Uox^-/-^ rats were mated with female wild-type rats.

Six nests of male Uox^-/-^ rats, two nests of male Uox^+/-^ rats, and two nests of male wild-type rats were enriched in the laboratory. The rats were maintained at 22°C, with a humidity of 45%–55% under natural light and a free approach to food and water. All animal experiments were approved by the Animal Care and Use Committee of Kunming Medical University (Approved No. KMMU-2020196) and performed under the Guidelines for Ethical Review of Laboratory Animal Welfare of China.

The living rats after the experiment were intraperitoneally anesthetized with urethane (1.0 g/kg). When they were under deep anesthesia, their necks were dislocated for euthanasia. The rat bodies had been collected in yellow plastic bags and kept in a refrigerator at –20°C until they were taken away by a green company for cremation.

### Animal experiment

When the male animals were 45 days old, they were weighed, and their SUA was assayed. When they were 45 days old or weighed 180–220 g, they were individually kept in metabolic cages. The amount of food and water they consumed during 24 h was recorded, and the feces and urine they excreted were also collected and recorded. Next, uric acid in feces and urine was assayed. After then, the animals were sacrificed, and their abdominopelvic and thoracic cavities were opened. Their organs, including the heart, liver, spleen, lung, kidney, thymus, adrenal gland, stomach, duodenum, and ileum, were harvested.

The gastrointestinal segments and juice were harvested in line with the methods described previously to determine uric acid in the tissues and the fluids [15].

Animal organs were weighed, and their organ indexes were used to evaluate the overall organ status between wild-type and Uox^-/-^ rats. The organ index was calculated using [Formula-1.


$$\mathrm{Organ}\;Index=\frac{weight_{\mathrm{organ}}}{weight_{body}}\times 100\%$$ (Formula-1)

### Collection of samples for uric acid determination

The rats were kept in small cages, a blood sample of about 0.2 ml was drawn from the tail vein using a tiny needle without anesthetization at a local atmosphere of 28°C–32°C. Serum was obtained by spinning at 3,000g and 4°C for 5 min as soon as the blood coagulated. Urine and feces were collected on ice in a cold insulation box. The urine was stirred to a homogeneous state, and 1.2 ml was piped as the original sample. The original sample was quickly diluted 20 times as the final sample for uric acid assay. The feces was weighed and mixed with three folds of 100 mmol/L Tris solution in weight, and the mixture was stirred at a shaker at 100 rpm for 4 h. The mixture was spun at 5000g and 4°C for 5 min, and the supernatant was collected as the sample for uric acid. All the samples for uric acid assay were kept at –20°C or determined as soon as possible to prevent the false elevation of uric acid levels by xanthine dehydrogenase [16]. Uric acid in the samples was assayed using the uric acid assay kit. The assay kit had a quantification range of uric acid from 3.91 μg/ml to 125 μg/ml. If uric acid in the sample was above the range, the sample was diluted. The protocol of uric acid assay kit is available at http://www.njjcbio.com/uploadfile/product/big/20190612093216738.pdf.

### BUN, Cr, blood lipids, and glucose determination

Serum samples used to evaluate SUA were also used to evaluate BUN, Cr, AST, ALT, Tbil, blood fats, and serum glucose. BUN (mmol/l) and Cr (nmol/l) in the serum samples were determined using the urea assay kits and creatinine assay kits, respectively, in accordance with the protocols provided by the producer. Blood lipid indexes, including TG, TC, LDL, and HDL in serum, serum glucose, AST, and ALT were determined using the assay kits in line with the protocols provided by the manufacturer. All the protocols are available at the following websites: http://www.njjcbio.com/uploadfile/product/big/20190612093249433.pdf for BUN assay; http://www.njjcbio.com/uploadfile/product/big/20190612093006383.pdf for Cr assay; http://www.njjcbio.com/uploadfile/product/big/20190611151410510.pdf for TG assay; http://www.njjcbio.com/uploadfile/product/big/20190611151511722.pdf for TC assay; http://www.njjcbio.com/uploadfile/product/big/20190611151926041.pdf for LDL assay; http://www.njjcbio.com/uploadfile/product/big/20190611151745050.pdf for HDL assay; http://www.njjcbio.com/products.asp?id=812 for glucose assay, http://www.njjcbio.com/uploadfile/product/big/20200826161559514.pdf for AST assay, and http://www.njjcbio.com/uploadfile/product/big/20190612090953151.pdf for ALT assay.

### Histological examination of organ sections

The rats were anesthetized with urethane (1.0 g/kg). Their abdominopelvic and thoracic cavities were opened, and normal saline solution for injection was injected into their left ventricle immediately. The perfusate was discharged via the right atrium by making a small hole using small scissors. After injection of normal saline solution (200 ml), 4% formaldehyde solution (200 ml) was injected. Then, the heart, liver, spleen, lung, kidney, thymus, stomach, duodenum, and the terminal ileum were harvested, fixed in the 4% formaldehyde solution for for more than 24 h, and subjected to hematoxylin-eosin (HE) staining. The organs were immersed in the 4% paraformaldehyde solution until a routine HE staining was conducted. Paraffin-embedded sections (20 μm) of the organs were cut. The sections were stained using an HE staining kit (Boster Biological Engineering Co., Ltd., Wuhan, China). Images were visualized using the fluorescence microscope in a light mode.

### Statistical analysis

Values were expressed as mean ± standard deviation (SD) or mean ± standard error (SE). If normal distribution of values was verified by the normality test (Shapiro-Wilk test), one-way analysis of variance (ANOVA) was performed to compare means between different groups. If there was a significance, post-hoc tests between every two groups were performed using S-N-K method (equal variances assumed) or Tamhane’s T2 method (equal variances not assumed). Otherwise, a nonparametric test for two independent samples (Mann-Whitney U model, two-tailed) was performed. The correlation of uric acid between in the Uox^-/-^ rats’ gastrointestinal tissue and their gastrointestinal juice was tested using Pearson’s correlation (two-tailed). Statistical significance was accepted at P < 0.05.

## Results

### Body weight of Uox^-/-^ rats at age of 45 days

**Fig 1:**
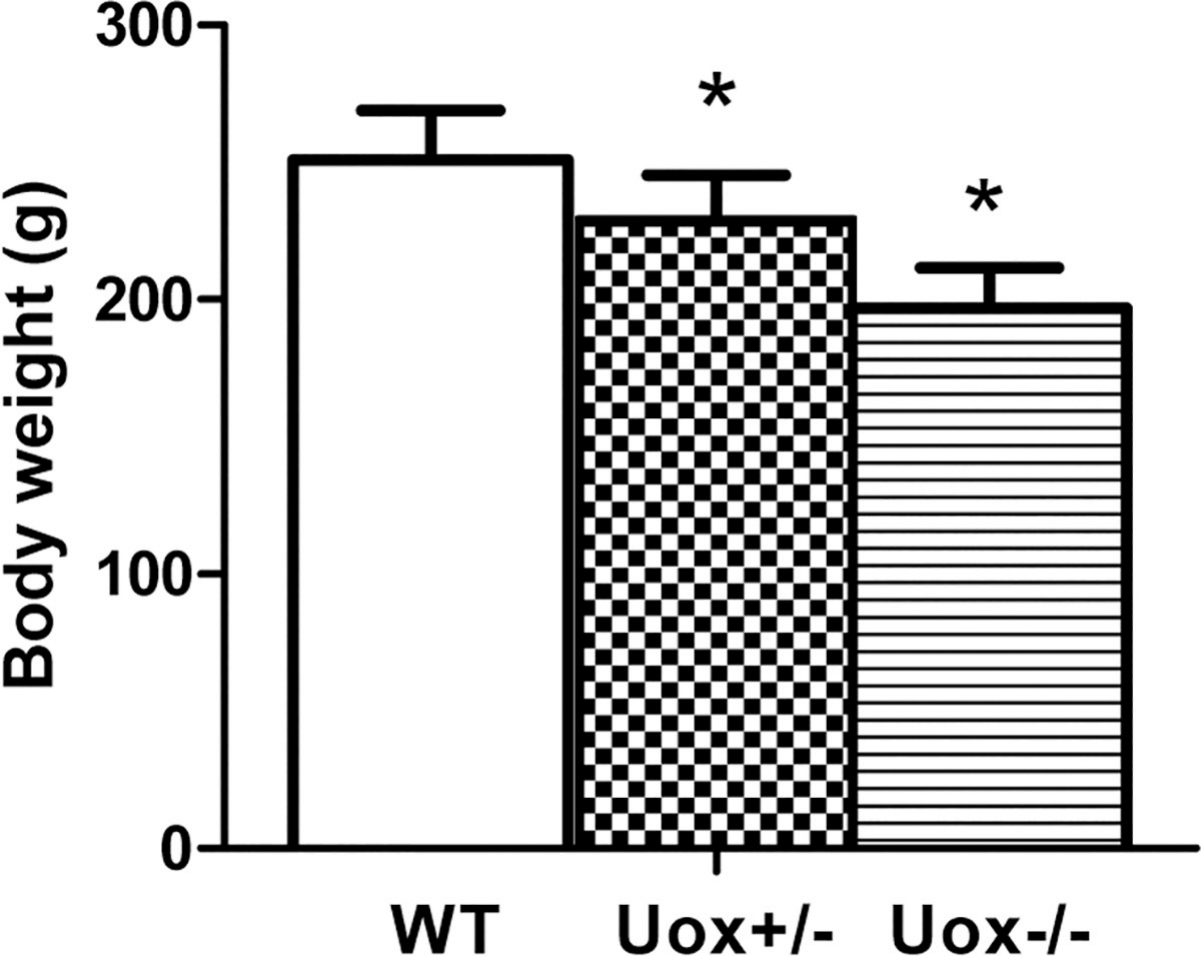
Body weight of the Uox^-/-^ rats at the age of 45 days (mean±SD, n = 6 (WT), or 10 (Uox^+/-^ and Uox^-/-^)). * P<0.05, vs Uox^+/-^ rats, one-way ANOVA.

The body weight of the male wild-type rats at the age of 45 days was the highest (250.8±18.0 g), and that of the Uox^-/-^ rats was the lowest (196.9 ± 14.8 g) (Fig 1). The results suggest that uricase deficiency retards the growth of rats.

### Dietary consumption and excretion of Uox^-/-^ rats

**Fig 2:**
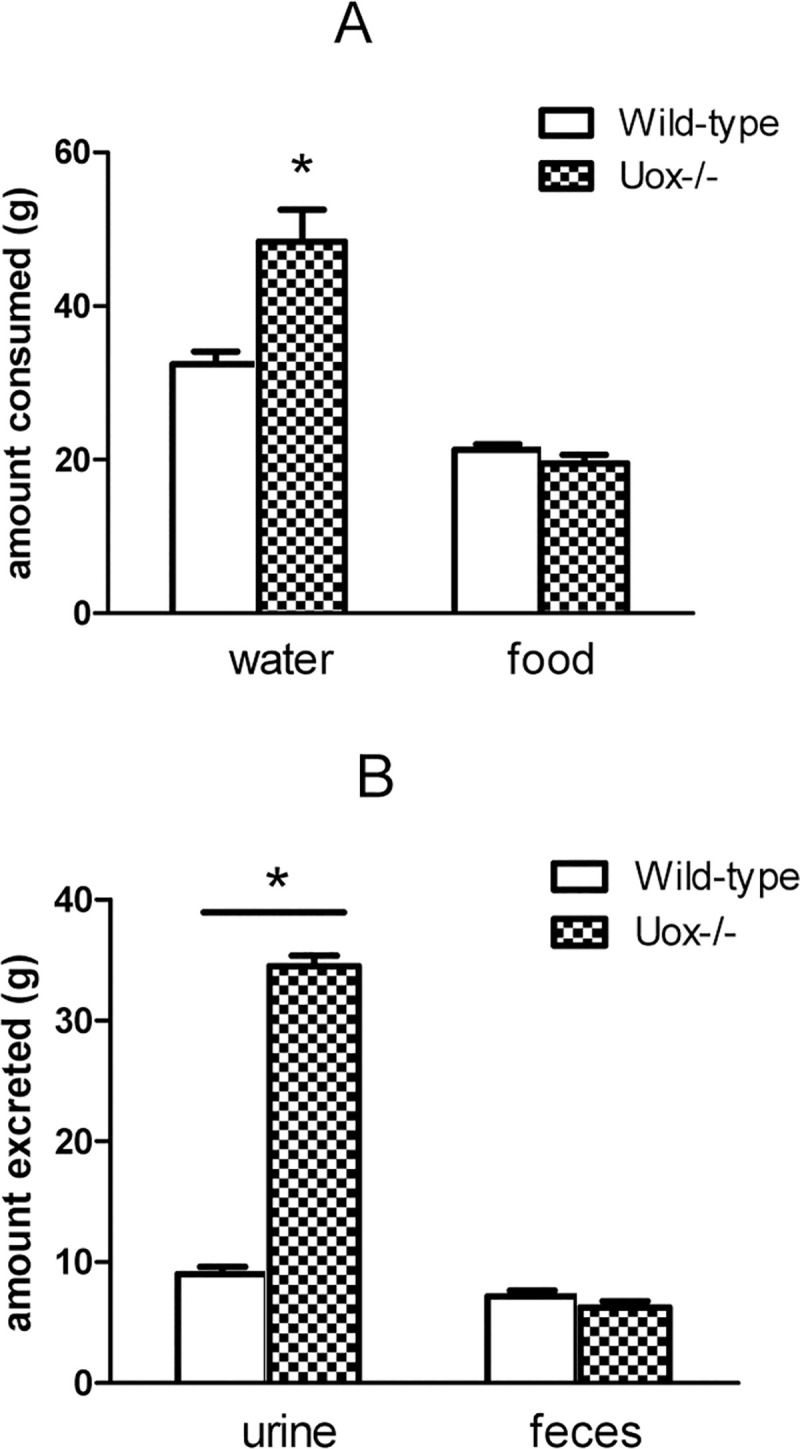
Dietary consumption and excretion of Uox^-/-^ rats at the age of 45 days (mean±SE, n = 10). A, the amounts of water and food consumed by the wild-type and Uox^-/-^ rats during 24 h. B, the amounts of urine and feces excreted by wild-type and Uox^-/-^ rats during 24 h. * P<0.05 vs wild-type, one-way ANOVA.

The male 45-day-old Uox^-/-^ rats consumed more water and excreted more urine during 24 h than the wild-type rats (Fig 2). However, the amounts of food consumed and feces excreted by the Uox^-/-^ rats were similar to those by wild-type rats (Fig 2). The increased urine excretion suggests renal injury Uox^-/-^ rats.

### Uric acid distribution in Uox^-/-^ rats

**Fig 3:**
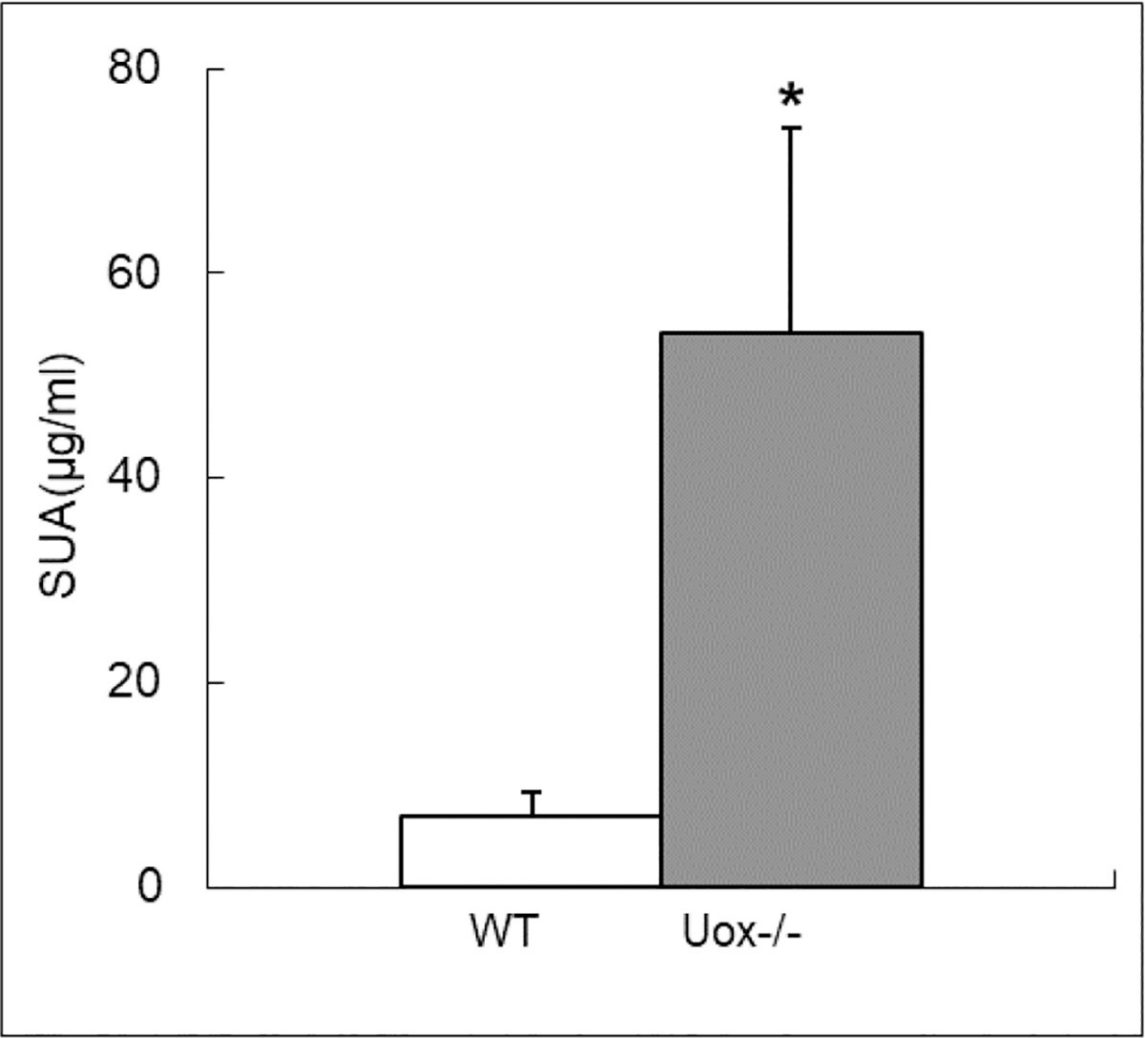
Serum Uric Acid (SUA) in the Wild-Type (WT) and Uox^-/-^ male rats (Uox^-/-^) weighing 180–220 g [mean±SD, n = 10 (WT) or = 11(Uox^-/-^)]. * P < 0.05, vs WT, one-way ANOVA.

The level of SUA in male Uox^-/-^ rats (180–220 g) was on average above 50 μg/ml, much higher than that in the wild-type rats of the same age (Fig 3), which is in agreement with our previous results [14].

**Fig 4:**
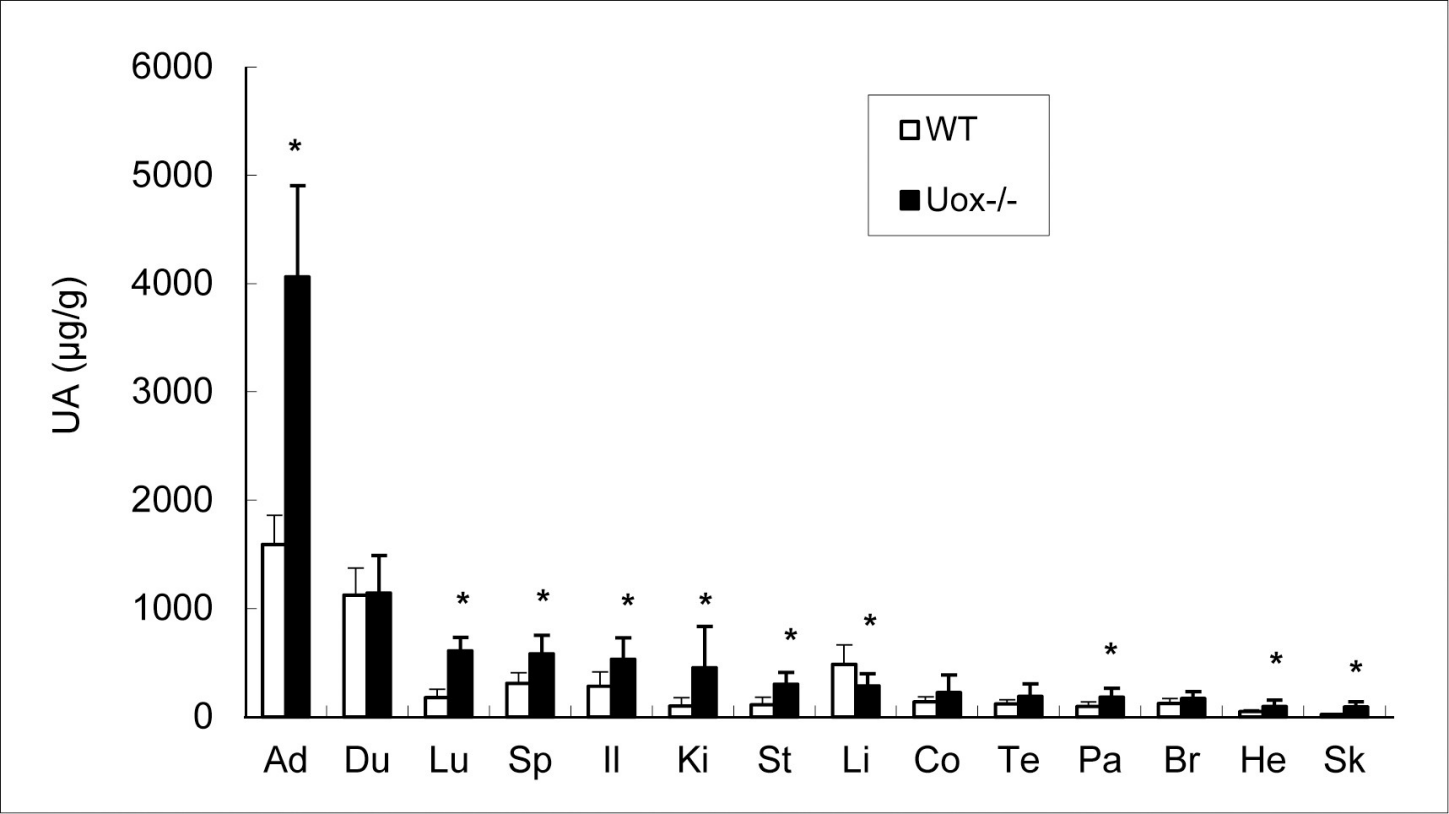
Uric Acid (UA) in the male Wild-Type (WT) and Uox^-/-^ rats’ tissues (Uox^-/-^) who weighing 180–220 g [mean±SD, n = 8 (WT) or = 10 (Uox^-/-^)]. * P < 0.05, vs WT, one-way ANOVA. Sorted by the uric acid level in Uox^-/-^ rats’ tissues from high to low. Ad, the adrenal gland; Du, the duodenum; Li, the liver; Sp, the spleen; Il, the ileum; Lu, the lung; Co, the colon; Br, the brain; Te, the testicle; St, the stomach; Ki, the kidney; Pa, the pancreas; He, the heart; Sk, the skeleton (gluteus).

Uric acid in the Uox^-/-^ rats’ organs was assayed. Except for the liver, uric acid content in the Uox^-/-^ rats’ organs was higher than that in the wild-type rats weighing 180–220 g (Fig 4). The top four organs with high uric acid distribution in Uox^-/-^ rats were the adrenal gland, duodenum, lung, and spleen; the top four organs according to uric acid content in wild-type rats were the adrenal gland, duodenum, liver, and ileum.

**Fig 5:**
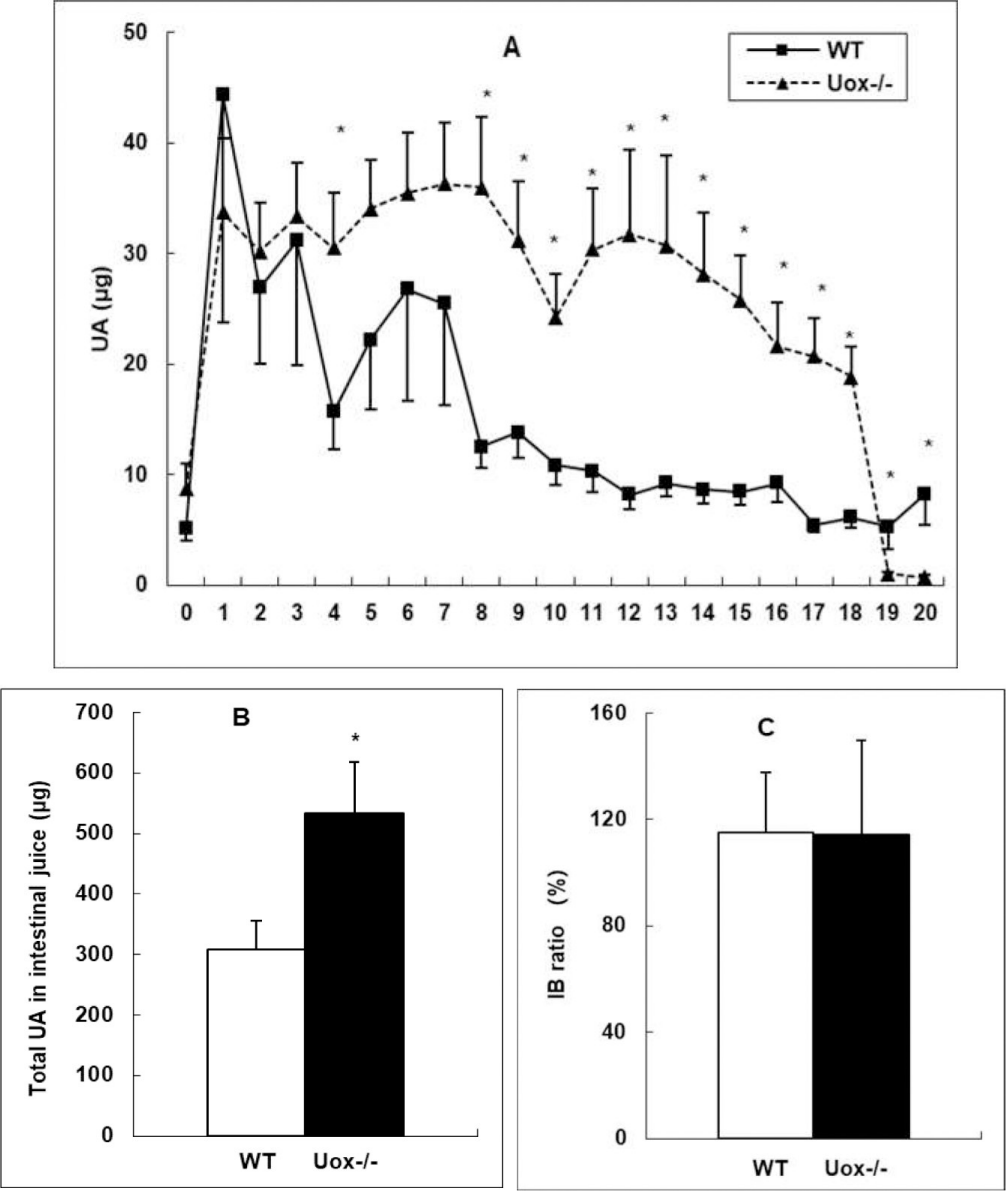
Uric Acid (UA) in the Wild-Type (WT) and Uox^-/-^ (Uox^-/-^) rats’ gastrointestinal juice who weighing 180–220 g (mean±SD, n = 10). A, Rats’ intestinal tract except cecum was equally divided into 20 segments, and uric acid in the intestinal juice was assayed. Segment 0 was the stomach, segment 1 was the duodenum, segments 2 to 7 were the jejunum, segments 8 to 18 were the ileum, and segments 19 to 20 were the colon. In all the groups, the uric acid distribution curve arrived to the maximum at segment 1, and almost declined to the terminus. Compared with the distribution curve of the WT group, those of the Uox^-/-^ group was almost parallelly increased to some extent. B, The total UA in intestinal juice in Uox^-/-^ group significantly increased. C, UA IB ratio in the Uox^-/-^ rats was similar to that in the WT rats. * P<0.05 vs WT, one-way ANOVA.

**Fig 6:**
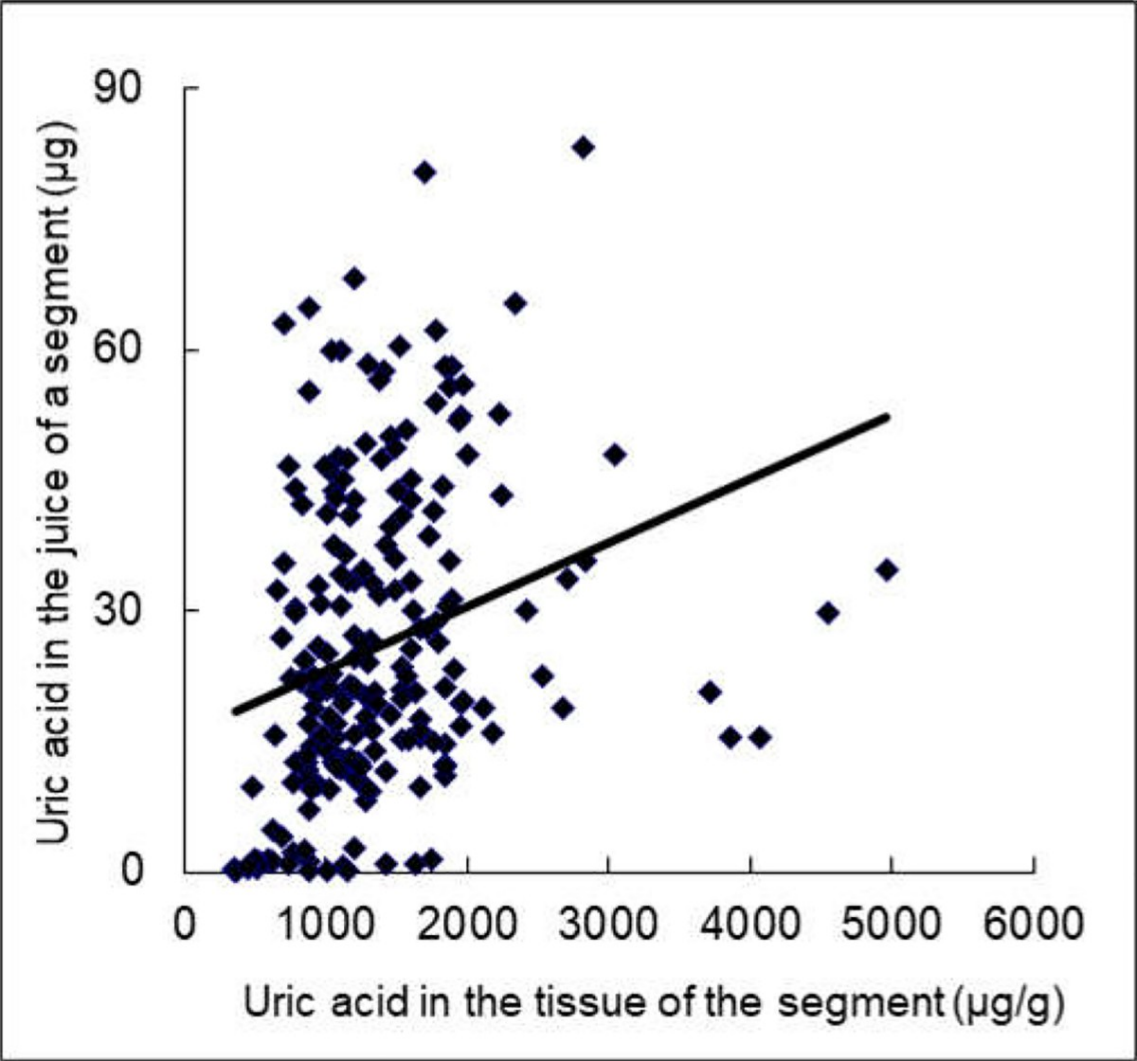
Correlation between Uric Acid (UA) in the Uox^-/-^ rats’ intestinal tissue and their gastrointestinal juice who weighed 180–220 g (n = 210). R = 0.274, P = 0.000, Pearson correlation (two-tailed).

**Fig 7:**
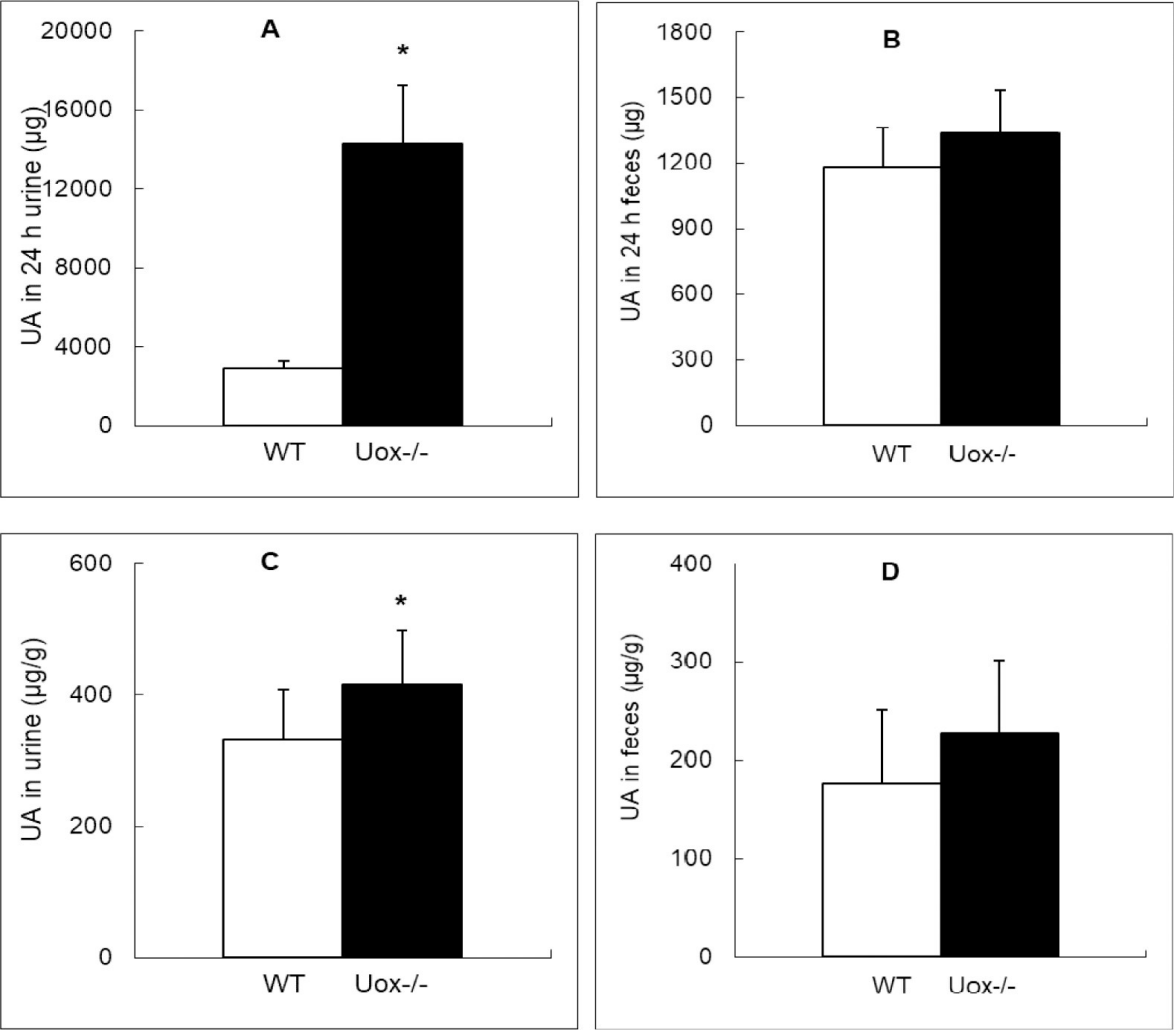
Uric Acid (UA) in the Wild-Type (WT) and Uox^-/-^ rats’ urine or feces who weighing 180–220 g (mean±SD). A, UA (μg) in the WT and Uox^-/-^ rats’ urine for 24 h (n = 10); B, UA (μg) in the WT and Uox-/- rats’ feces for 24 h (n = 10); C, UA content (μg/g) in the WT and Uox^-/-^ rats’ urine (n = 10); D, UA content (μg/g) in the WT and Uox^-/-^ rats’ feces (n = 10). * P<0.05, vs WT, one-way ANOVA.

Since the intestinal tract is one of the important sites of uric acid distribution [15], uric acid was assayed in gastrointestinal tissues and juice. The content of uric acid in the gastrointestinal fluid was higher in the Uox^-/-^ rats than in wild-type rats, except for the juice of the colon (segments 19 and 20) (Fig 5A). The uric acid distribution pattern in the Uox^-/-^ rats’ gastrointestinal juice was largely similar to that in wild-type rats’ [15], since the IB ratio (total uric acid in intestinal juice: by total uric acid in the blood; calculated by Formula-1) in the Uox^-/-^ rats was close to that in the wild-type rats (Fig 5C), though the total uric acid in Uox^-/-^ rats’ intestinal juice was much higher than that in the wild-type rats (Fig 5B). The uric acid content in the intestinal juice of a segment significantly correlated with that in the segment’s tissue (R = 0.274, P = 0.000) (Fig 6), suggesting that the level of uric acid in the juice of a segment was at least partly dependent on the tissue nearby. Compared with the wild-type rats, the total uric acid excreted through 24-h urine by the Uox^-/-^ rats was significantly higher (Fig 7A). However, the total uric acid excreted through feces by the Uox^-/-^ rats was not significantly higher (Fig 7B). The uric acid content was significantly higher in Uox^-/-^ rats’ urine (Fig 7C), but not significantly in Uox^-/-^ rats’ feces (Fig 7D).

### Serum indexes associated with kidney and liver function in the Uox^-/-^ rats

Hyperuricemia may be associated with renal function impairment, as well as with glucose and lipid metabolism disorders [2, 3]. BUN and Cr both increased in the Uox^-/-^ rats (Table 1), suggesting renal injury. The Uox^-/-^ rats tended to develop hyperglycemia and hyperlipidemia because glucose (Glu) and total cholesterol (TC) increased, though without achieving significance (Table 1). The liver function indexes, including AST, ALT, and Tbil, were higher in 45-day-old Uox^-/-^ rats. However, the indexes of the Uox^-/-^ rats were mainly in the normal range, but some indexes (BUN, Cr, AST, ALT, Tbil, and HDL) significantly varied compared with those of wild-type rats.

**Table 1:** Serum indexes associated the kidney and liver function in rats at the age of 45 days were assayed (mean±SD, n = 10).

Item	Wild-type (n = 6)	Uox^-/-^ (n = 12)	Student-t test (two-tailed)
SUA (μg/mL)	15.00±5.48	56.18±10.84	0.000
BUN (mmol/L)	7.17±1.00	10.55±1.46*	0.000
Cr (nmol/L)	31.83±2.93	39.18±2.86*	0.000
AST (u/L)	116.3±19.47	264.458±100.96*	0.003
ALT (u/L)	40.43±9.21	69.25±10.08*	0.000
Tbil (μmol/L)	1.755±0.12	1.87±0.25	0.331
Glu (mmol/L)	5.65±1.50	6.77±1.73	0.186
TG (mmol/L)	0.98±0.20	0.80±0.28	0.172
TC (mmol/L)	1.76±0.12	1.87±0.25	0.331
LDL (mmol/L)	0.64±0.12	0.55±0.13	0.190
HDL (mmol/L)	1.34±0.10	1.75±0.21*	0.000

SUA, serum uric acid; BUN, blood urea nitrogen; Cr, serum creatinine; AST, Aspartate aminotransferase; ALT, alanine aminotransferase; Tbil, total bilirubin; TG, triglyceride; TC, total cholesterol; LDL, low-density lipoprote

### Organ indexes of the Uox^-/-^ rats

**Fig 8:**
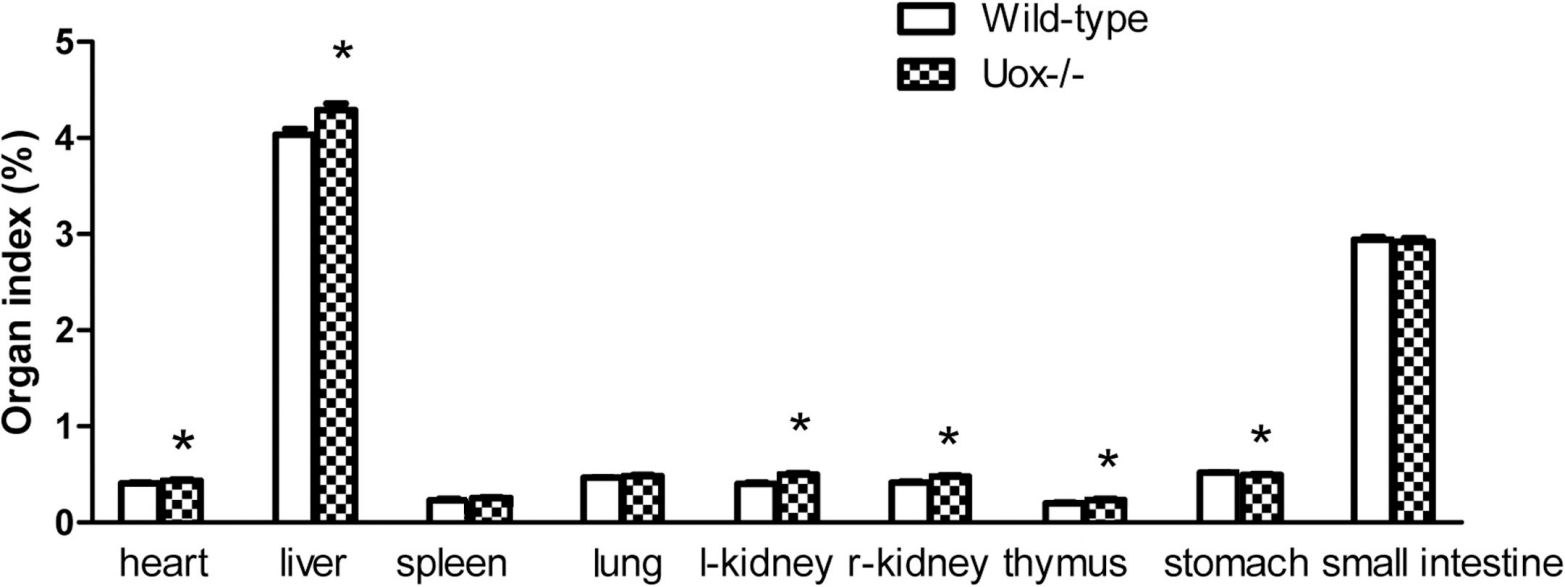
Organ indexes of the Uox^-/-^ rats weighing 180–220 g (mean±SE, n = 9). * P<0.05, vs the wild-type rats, one-way ANOVA.

**Fig 9:**
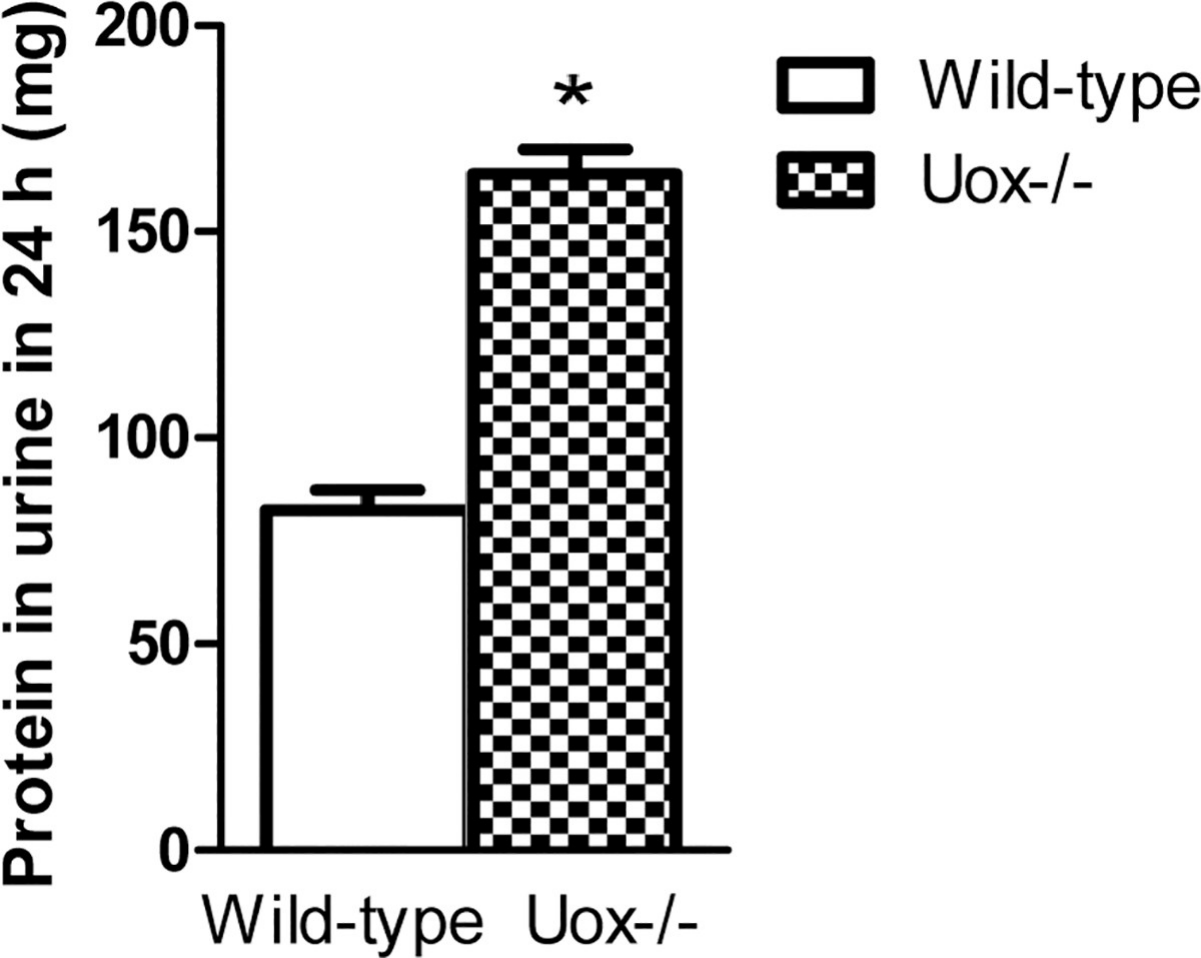
Protein in the urine excreted by the Wild-Type (WT) and Uox^-/-^ rats in 24 h who weighing 180–220 g (mean±SD, n = 10). * P<0.05, vs the wild-type rats, one-way ANOVA.

In the Uox^-/-^ rats weighing 180–220 g, most of their organ indexes tended to increase to some extent (Fig 8). Their heart, liver, kidney, and thymus indexes significantly increased, while their spleen and lung indexes increased without reaching significance. However, their stomach index significantly decreased, and the small intestine index decreased though without reaching statistical significance. The results suggested that parenchymatous organs tended to swell or accrete, whereas cavity organs tended to shrink or atrophy. Furthermore, the correlative analysis between the level of SUA and organ indexes verified that the increase in SUA was mainly associated with the damage to the liver, kidney, and stomach (Table 2). The increase in SUA or uricase deficiency resulted in apparent renal damage, considering that the amount of urine excreted by the Uox^-/-^ rats was almost three times as high as that excreted by the wild-type rats (Fig 2B). The amount of protein in the 24-h urine excreted by the Uox^-/-^ rats was almost twice as high as that excreted by the wild-type rats (Fig 9). In addtion, the increase in SUA negatively correlated with body weight (Table 2), which agrees with the results shown in Fig 1 and our previous report [17].

**Table 2:** Correlation between SUA and organ indexes in rats weighing 180–220 g (n = 18).

	heart	liver	spleen	lung	left kidney	right kidney	thymus	stomach	Small intestine	Body Weight
Pearson’s Correlation	.464	.515*	.361	.295	.813*	.762*	.416	-.514*	-.063	-.476*
Sig. (2-tailed)	.052	.029	.141	.235	.000	.000	.086	.029	.803	.046

### Histological examination of organ sections in the Uox^-/-^ rats

**Fig 10:**
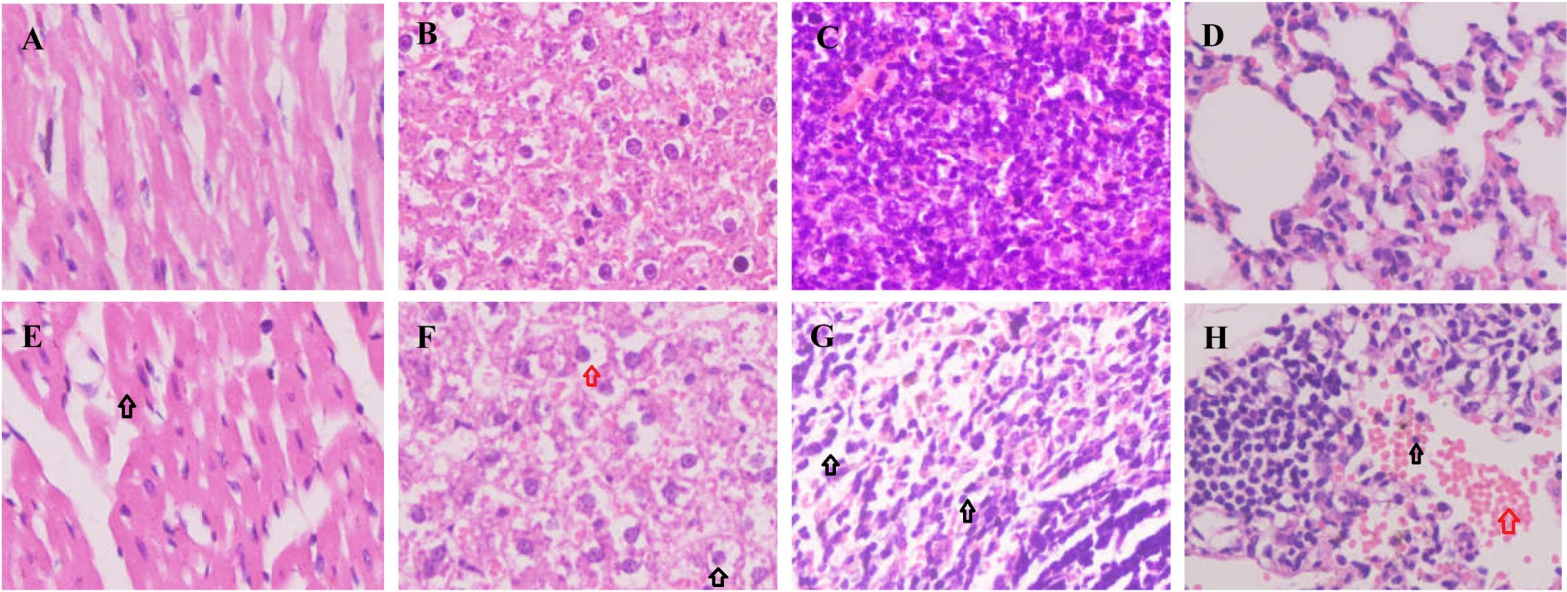
Histological examination of the heart, liver, spleen, and lung sections. Organ sections were stained with hematoxylin-eosin (HE). Organ sections were made and stained with HE. Mildly swollen cardiomyocytes (E, the black arrow) and hepatocytes (F, the black arrow) were found in the Uox^-/-^ rats’ heart sections and liver sections, respectively. There could be fat accumulation in the hepatocytes’ cytoplasm, because the vacuolation usually made by fats was seen near the nucleus (F, the red arrow). The decreased splenocytes and the increased interstitial tissues (G, the black arrows) were also found in the Uox^-/-^ rats’ spleen sections. In the Uox^-/-^ rats’ lung sections (H), exudative inflammatory cells (the black arrow) and even red blood cells (the red arrow) were seen. A, the heart of the wild-type rat; B, the liver of the wild-type rat; C, the spleen of the wild-type rat; D, the lung of the wild-type rat; E, the heart of the Uox^-/-^ rat; F, the liver of the Uox^-/-^ rat; G, the spleen of the Uox^-/-^ rat; and H, the lung of the Uox^-/-^ rat. Picture = 130 μm × 175 μm.

The organs were collected from rats weighing 180–220 g. Organ sections were made and stained with HE. Mildly swollen cardiomyocytes (Fig 10E) and hepatocytes (Fig 10F) were found in the Uox^-/-^ rats’ heart sections and liver sections, respectively. There could be fat accumulation in the hepatocytes’ cytoplasm because the vacuolation usually made by fats was seen near the nuclei (Fig 10F). The decreased splenocytes and the increased interstital tissues (Fig 10G) were also found in the Uox^-/-^ rats’ spleen sections. In the Uox^-/-^ rats’ lung sections (Fig 10H), exudative inflammatory cells, and even red blood cells were seen.

**Fig 11:**
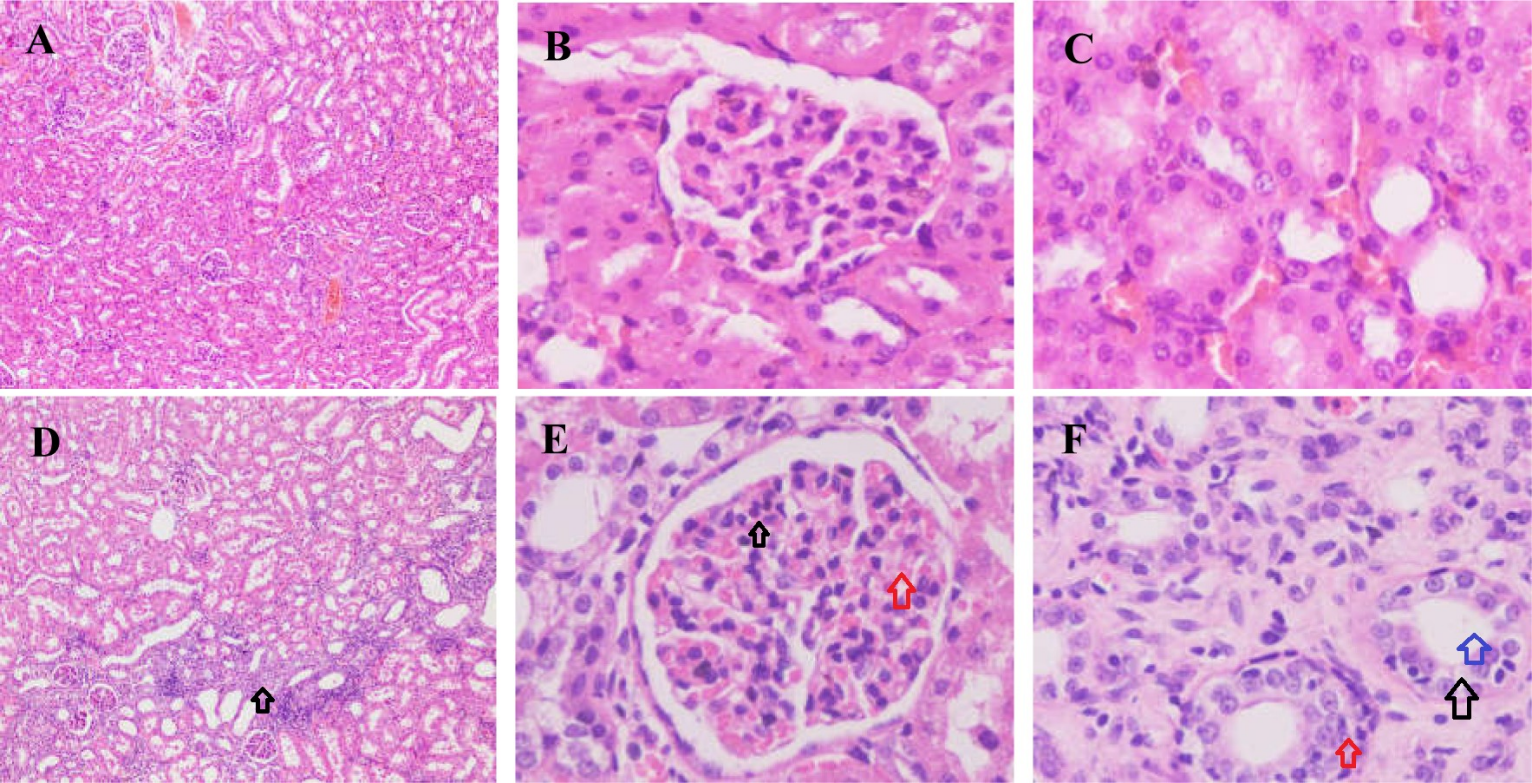
Histological examination of the kidney sections. Organ sections were stained with hematoxylin-eosin (HE). Interstitial hyperplasia was frequently found (D, the black arrow). The swollen glomeruli, the thronged erythrocytes (the red arrow) in the glomeruli, and the proliferated glomerular cells (the black arrow) were often found (E) in the Uox^-/-^ rats’ kidney sections. The enlarged tubules (the blue arrow), swollen tubular walls (the red arrow) and proliferated renal cells (the black arrow) were also frequently seen in the Uox^-/-^ rats’ renal interstitial tissue (F). A-C, the kidney of the wild-type rat and D-E, the kidney of the Uox^-/-^ rat. Picture (A and D) = 1040 μm × 1400 μm; and Picture (B, C, E, and F) = 130 μm × 175 μm.

As mentioned above, the increased SUA or uricase deficiency impaired the renal function. In the Uox^-/-^ rats’ kidney sections (Fig 11D), interstitial hyperplasia was frequently found. The swollen glomeruli, the thronged erythrocytes in the glomeruli, and the proliferated glomerular cells were also frequently observed (Fig 11E) in the Uox^-/-^ rats’ kidney sections. The enlarged tubules, swollen tubular walls, and proliferated renal cells were commonly seen in the Uox^-/-^ rats’ renal interstitial tissue (Fig 11F).

**Fig 12:**
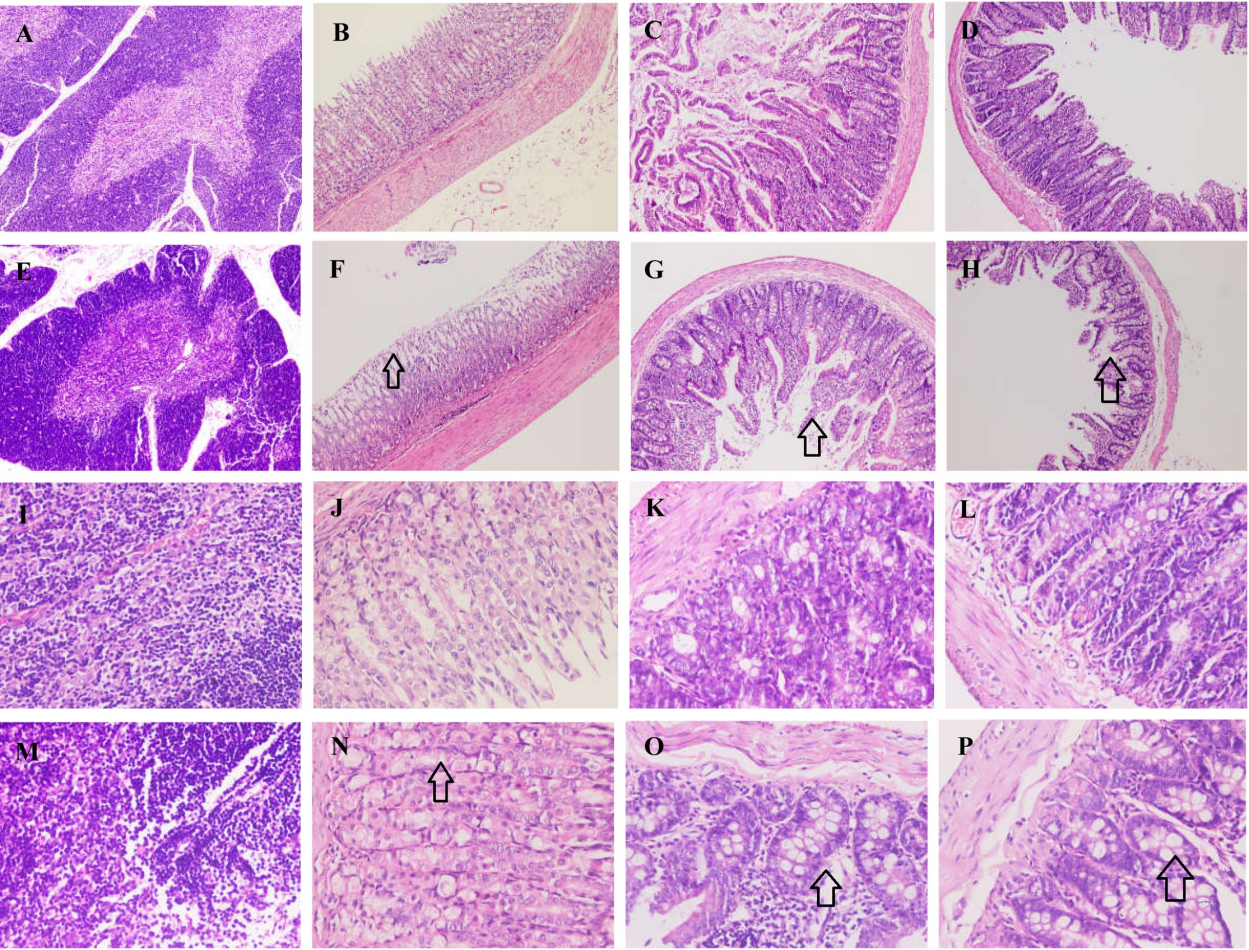
Histological examination of the thymus, stomach, duodenum, and ileum sections. Organ sections were stained with hematoxylin-eosin (HE). The thymocytes increased in the Uox^-/-^ rats’ thymus (E and M), while the villi of the gastric mucosa in the Uox^-/-^ rats’ stomach (F and N), duodenum (G and O), and ileum (H and P) were shortened (the black arrow in F, G, and H) and swollen (the black arrow in N, O, and P). A, the thymus of the wild-type rat; B, the stomach of the wild-type rat; C, the duodenum of the wild-type rat; D, the ileum of the wild-type rat; E, the thymus of the Uox^-/-^ rat; F, the stomach of the Uox^-/-^ rat; G, the duodenum of the Uox^-/-^ rat; H, the ileum of the Uox^-/-^ rat; I, the thymus of the wild-type rat; J, the stomach of the wild-type rat; K, the duodenum of the wild-type rat; L, the ileum of the wild-type rat; M, the thymus of the Uox^-/-^ rat; N, the stomach of the Uox^-/-^ rat; O, the duodenum of the Uox^-/^- rat; and P, the ileum of the Uox^-/-^ rat. Picture(A-H) = 1040 μm × 1400 μm; Picture (I-P) = 260 μm × 350 μm.

As for other organs, the thymocytes increased in the Uox^-/-^ rats’ thymus (Fig 12E and 12M), while the villi of the gastric mucosa in the Uox^-/-^ rats’ stomach (Fig 12F and 12N), duodenum (Fig 12G and 12O), and ileum (Fig 12H and 12P) were shortened and swollen.

## Discussion

Theoretically, compared with Uox^-/-^ mice [12, 13], Uox^-/-^ rats could be one of the optimal model animals to study hyperuricemia and associated diseases because the animals’ purine metabolism is similar to that of men [14]. The Uox^-/-^ showed some advantages to evaluate the urate-lowering effect of some drugs [18, 19]. However, before the animals can be accepted for general use, their biological properties should be elucidated. Considering that hyperuricemia and associated diseases more often occur in men than in women, male rats were selected in the present study.

### Uricase deficiency retards growth and causes multiple injuries

The present study showed that the male Uox^-/-^ rats’ growth was retarded (Fig 1). The young male Uox^-/-^ rats consumed a similar amount of food to the wild-type rats of the same age and excreted an equivalent amount of feces (Fig 2). However, the Uox^-/-^ rats drunk more water and excreted more urine, suggesting a renal dysfunction resulting from uricase deficiency (Fig 2).

As expected, BUN and Cr, indexes related to renal function injury, significantly increased (Table 1), though the values were still in the normal ranges. Considering the excretion of BUN and Cr is mainly associated with glomerular filtration rate and the amount of urine in the Uox^-/-^ is mostly related to tubular reabsorption, the renal injury may involve both glomeruli and tubules. Indeed, the mild injuries in the glomeruli, tubule, and interstitial tissues were verified by histological examination (Fig 11). The renal injury results from uricase deficiency rather than from the increased SUA, while injuries of other organs are more likely caused by the increased SUA [12, 13]. However, polyuresis in the Uox^-/-^ rats was observed, suggesting early or mild renal tubular injury.

Hyperuricemia usually correlates with dyslipidemia [2] (Mortada, 2017). However, the present study did not find the differences in TG, TC, and LDL between the Uox^-/-^ and wild-type rats. To our surprise, HDL, which is believed to be a “good” lipoprotein, was significantly increased. This finding is different from that in hyperuricemic rats caused by high-purine diet or potassium oxonate (a uricase inhibitor) [8]. AST and ALT are enzymes mainly expressed in hepatocytes, and they can escape to blood if the hepatocytes’ membrane is broken. The two enzymes in serum are widely used to diagnose liver damage. The increase in AST and ALT (Table 1) suggested that the membranes of hepatocytes in the Uox^-/-^ rats were fragile.

As mentioned above, uricase deficiency retarded rat growth. However, the deficiency increased the parenchymatous organs’ indexes, including the heart, liver, kidney, and thymus, but decreased the cavity organs’ indexes, such as the stomach and small intestine (Fig 8). The significantly changed organ indexes indicated that there could be some disorder at the histological level. The histological examination (Figs 10–12) proved the existence of disorders. Among them, similar intestinal injury was previously observed in Uox^-/-^ mice [4].

### Reasonable mechanism for multiple injuries in Uox^-/-^ rats

The multiple organs’ injuries could result from uricase deficiency or the increase in SUA. According to the early report, Uox^-/-^ mice had an extremely high level of SUA [12, 13], which quickly caused high mortality because of the sediment of uric acid in the kidney. Urate crystals were found in their renal interstitium under polarized light [13]. In contrast to Uox^-/-^ mice, the SUA level in Uox^-/-^ rats was much lower, only similar to that in humans, and the urate crystals were not found in their kidneys. The results are understandable since urate crystals are seldom found in normal persons whose SUA level is similar to that of the Uox^-/-^ rats. Furthermore, the uric acid content in the wild-type rats’ adrenal glands was higher than that in their other organs, so was in Uox^-/-^ rats (Fig 4). However, no reports have shown the urate crystals in wild-type rats’ adrenal glands. Considering that uricase is almost only expressed in the rat liver [14], the organ injuries resulted more likely from the increase in SUA than the direct uricase deficiency. The results of multiple organ injuries also suggested that different organs have different sensitivities to the increase in SUA. As for Uox^-/-^ rats, their liver injury occurred despite the level of uric acid content compared with that in wild-type rats (Fig 4); this suggests that the abridged transcript of Uox mRNA might play a role in liver damage because the abridged Uox gene is transcribed and spliced with a different pattern [14]; however, the exact mechanism needs further investigation.

### The balance between SUA level and uric acid excretion

Uricase deficiency increased SUA and even caused hyperuricemia in Uox^-/-^ rats (Fig 3), leading to the increased uric acid excretion in urine (Fig 7) and the tendency to the increased uric acid excretion via feces. Moreover, uric acid in the intestinal juice also significantly increased in the Uox^-/-^ rats. Uric acid in the upper intestinal fluid was higher than that in the lower intestinal fluid. Like in the wild-type rats [15], the distribution pattern in the Uox^-/-^ rats may also be associated with uric acid synthesis and secretion in the upper intestinal tract and the degradation and reclamation in the lower intestinal tract. Especially, the uric acid in the intestinal tissue significantly correlated with that in the intestinal juice nearby (Fig 6). It is reasonable to believe that the uric acid in the intestinal juice was affected by the tissue nearby. Because the uric acid IB ratio in the Uox^-/-^ rats was similar to that in the wild-type rats (Fig 5), it is reasonable to deduce that the SUA level in Uox^-/-^ rats can be lowered via intestinal tract like in the wild-type rats [15].

It should be noted that uricase deficiency is a crucial factor that accounts for the increase of SUA in Uox^-/-^ rats. It is taken for granted that there could be a negative feedback to neutralize the increased SUA level caused by uricase deficiency. In the present study, no factors (like Xdh expression) associated with the upregulation of uric acid synthesis were found. The feedback was based on the increase of uric acid excretion in urine, because the uric acid concentration in Uox^-/-^ rats’ urine and the total uric acid excreted through their urine were both higher than those in the wild-type rats’ urine (Fig 7). The intestinal tract could also contribute to the feedback because the uric acid content in Uox^-/-^ rats’ feces and the total uric acid excreted through their feces tended to be higher than those in the wild-type rats (Fig 7). Indeed, if uric acid in the rats’ intestinal tract was removed by a laxative, such as Paidu Yangyan capsule, the SUA level would be lowered [18]. The intestinal tract has been shown to be effective in lowering the SUA level in pigs [20]. Considering that the intestinal tract is a dominant place where many bacteria reside and some strategies of lowering SUA are based on the gut microbiome [21], gut bacteria could affect SUA. According to the recent reports, the bacterial flora in the male uricase-deficient rats [19] (Fan et al., 2021) or in the male “hyperuricemic” rats [21] was changed. Since uricase is almost universally expressed in microorganisms [22], it is taken for granted that gut microbes play a vital role in lowering the SUA levels. However, the aboriginal microbiome has not been proven to be the main factor affecting SUA [19], given that the combination of ampicillin and ciprofoxacin decreased rather than increased SUA level in the uricase-deficient rats.

## Conclusions

Uricase deficiency is harmful to rats’ health. Uricase deficiency retards the growth of young male rats, and the subsequent increase in SUA causes mild injuries to their kidneys, liver and other organs.

## Supporting information

S1 Fig (XLSX)

S2 Fig (XLSX)

S3 Fig (XLS)

S4 Fig (XLS)

S5 Fig (XLS)

S6 Fig (XLS)

S7 Fig (XLS)

S8 Fig (XLSX)

S9 Fig (XLSX)

S10 Fig (JPG)

S11 Fig (JPG)

S12 Fig (JPG)

S1 Table (XLS)

S2 Table (XLSX)
