# Supplementary figures and images for: Uricase deficiency causes mild and multiple organ injuries in rats
Source: PLoS One. 2021 Aug 26;16(8):e0256594. doi: 10.1371/journal.pone.0256594 (PMC8389383; doi:10.1371/journal.pone.0256594)

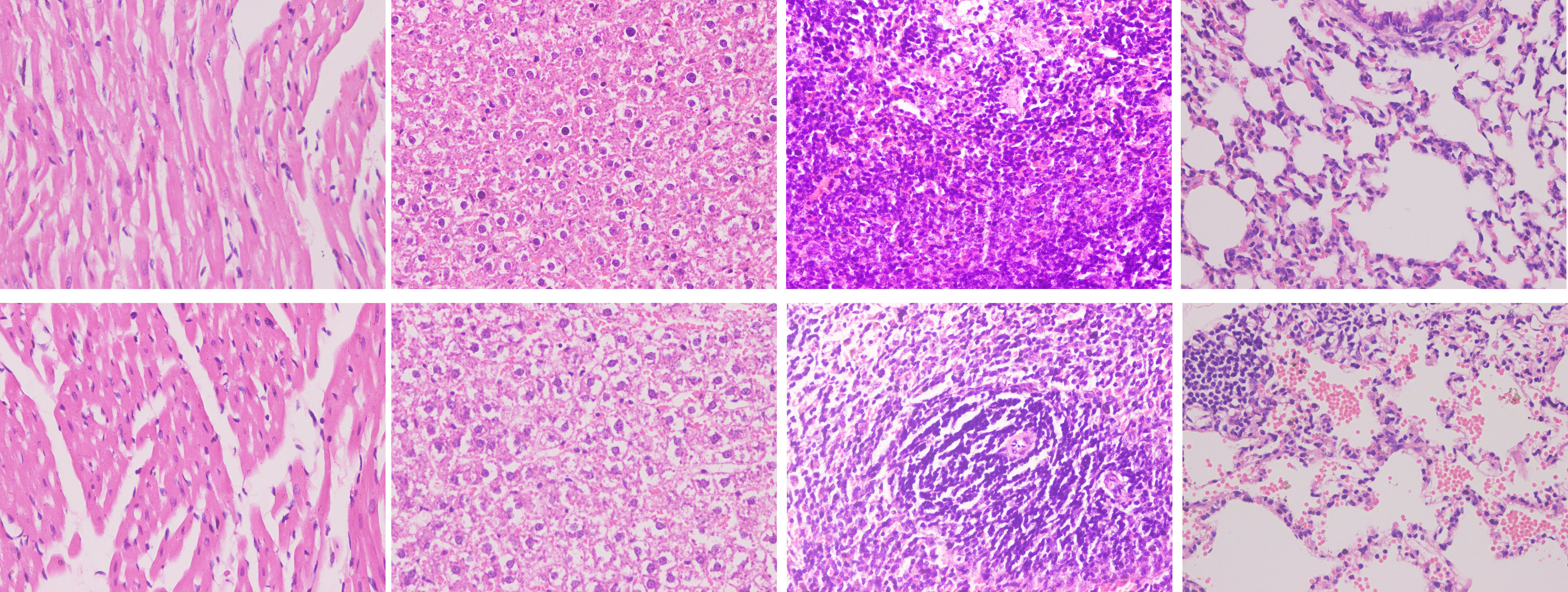

Supplement: S10 Fig — (JPG) [file pone.0256594.s010.jpg]

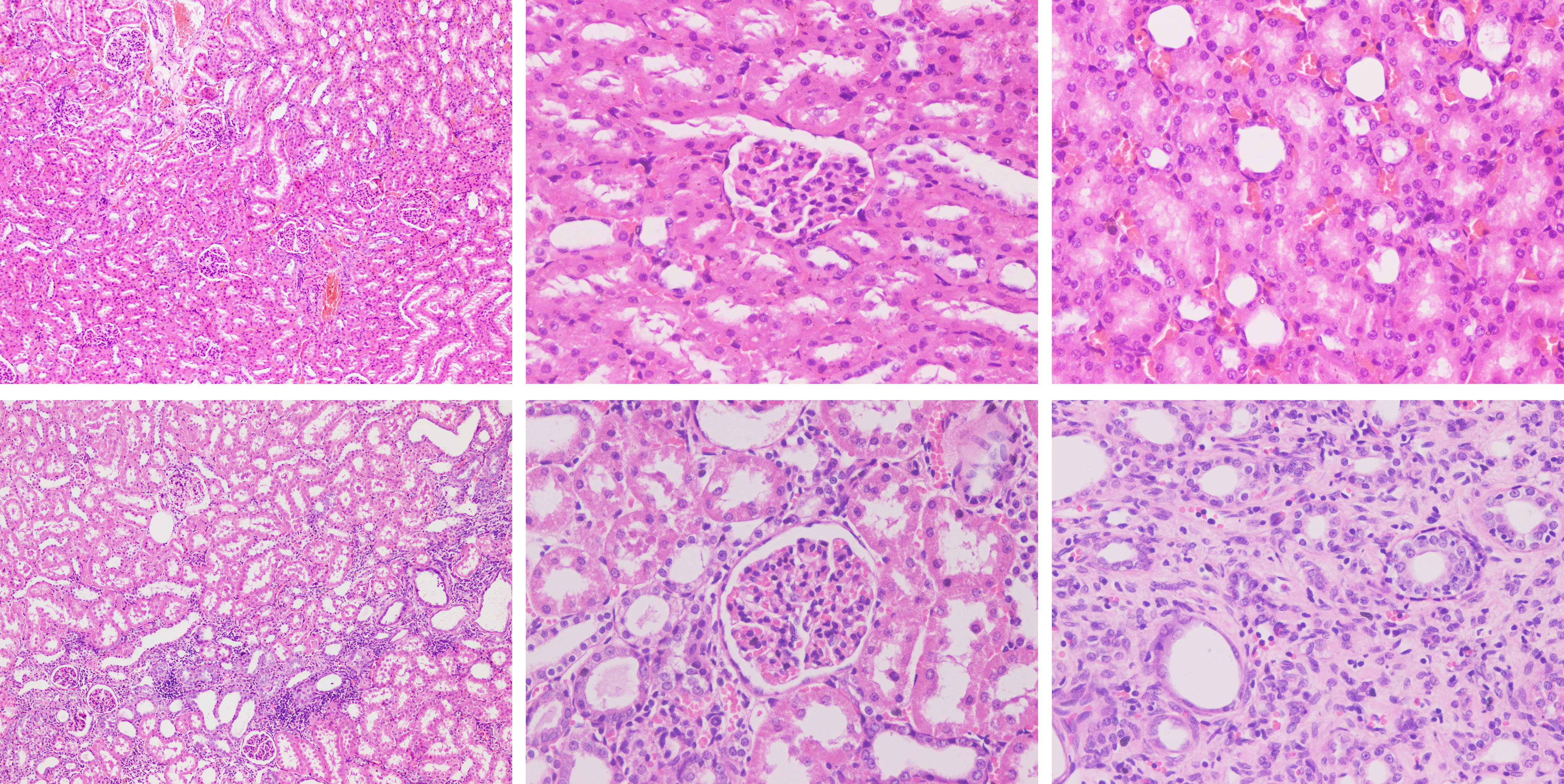

Supplement: S11 Fig — (JPG) [file pone.0256594.s011.jpg]

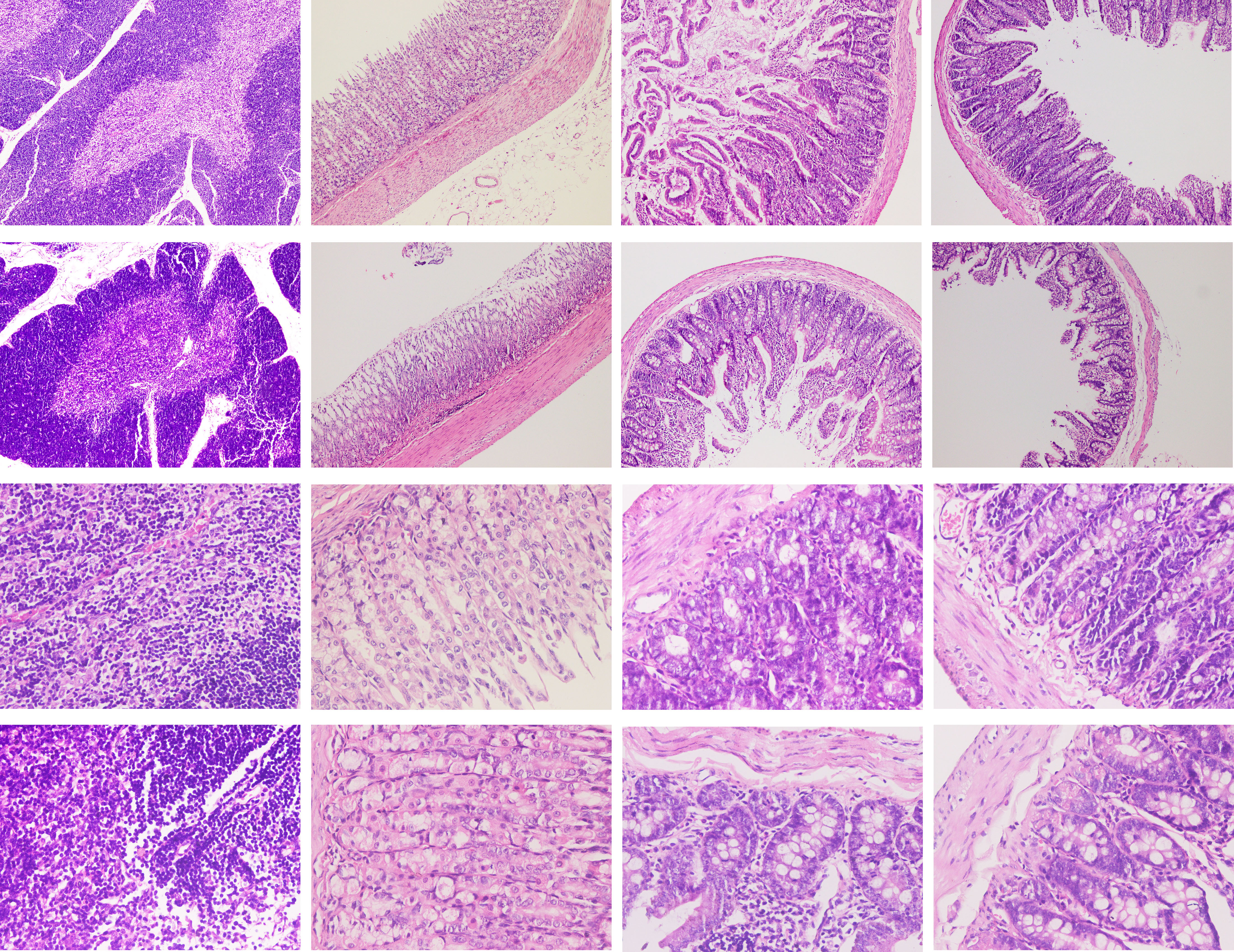

Supplement: S12 Fig — (JPG) [file pone.0256594.s012.jpg]
